# Impact of recent climate change on corn, rice, and wheat in southeastern USA

**DOI:** 10.1038/s41598-022-21454-3

**Published:** 2022-10-08

**Authors:** Ramandeep Kumar Sharma, Sunny Kumar, Kamal Vatta, Raju Bheemanahalli, Jagmandeep Dhillon, Krishna N. Reddy

**Affiliations:** 1 Department of Plant and Soil Sciences, Mississippi State University, Mississippi, USA; 2 Punjab Agricultural University, Ludhiana, Punjab India; 3 Crop Production Systems Research Unit, USDA-ARS, Stoneville, MS USA

**Keywords:** Plant sciences, Climate sciences

## Abstract

Climate change and its impact on agriculture productivity vary among crops and regions. The southeastern United States (SE-US) is agro-ecologically diversified, economically dependent on agriculture, and mostly overlooked by agroclimatic researchers. The objective of this study was to compute the effect of climatic variables; daily maximum temperature (T_max_), daily minimum temperature (T_min_), and rainfall on the yield of major cereal crops i.e., corn (*Zea mays* L.), rice (*Oryza sativa* L.), and wheat (*Triticum aestivum* L.) in SE-US. A fixed-effect model (panel data approach) was used by applying the production function on panel data from 1980 to 2020 from 11 SE-US states. An asymmetrical warming pattern was observed, where nocturnal warming was 105.90%, 106.30%, and 32.14%, higher than the diurnal warming during corn, rice, and wheat growing seasons, respectively. Additionally, a shift in rainfall was noticed ranging from 19.2 to 37.2 mm over different growing seasons. Rainfall significantly reduced wheat yield, while, it had no effect on corn and rice yields. The T_max_ and T_min_ had no significant effect on wheat yield. A 1 °C rise in T_max_ significantly decreased corn (− 34%) and rice (− 8.30%) yield which was offset by a 1 °C increase in T_min_ increasing corn (47%) and rice (22.40%) yield. Conclusively, overall temperature change of 1 °C in the SE-US significantly improved corn yield by 13%, rice yield by 14.10%, and had no effect on wheat yield.

## Introduction

Climate change is characterized as substantial long-term shifts in meteorological parameters such as temperature and rainfall^1–3^. Changing climate is an inevitable phenomenon and its effects are felt across the universe^4^. This is alarming considering variations in meteorological parameters impact crop production^5^. This is even more concerning considering that cereal production needs to be increased by 70–100% to ensure food security for the 9.8 billion people by 2050^6^. Cereals provide largest number of calories and nutrients to humans and animals, hence, cover most area than any other crop^7^. Human intervention via the use of fossils, deforestation, and land-cover alteration, lead to increased production of greenhouse gases, which is the main cause of global temperature increase^8,9^. Furthermore, throughout the twenty-first century, the duration and intensity of drought have become severe, reducing agricultural water reserves fivefold^6^. Unabated, global average temperature is expected to rise by 1.50 °C through 2050^10^. By the end of the twenty-first century, this increase could be as much as 3–5 °C at certain locations^9^. Moreover, the pace of global climate change over the next 20–70 years is expected to be more rapid and intense than in the previous 10,000 years^11,12^.

The shifting climate constitutes increases in nocturnal and diurnal warming along with irregular rainfall patterns^13^. Changes in these factors impact cereal production directly via inducing abiotic stresses^14^ and indirectly via biotic stresses such as insect and weed pests’ pressure, decreased beneficial soil microorganism community, etc.^15^. Increasing temperature reduces yield by reducing the grain filling period^16^. Extreme temperatures during the blooming stage also reduce cereal kernel count, thickness, and quality^17,18^. Timely rainfall could mitigate rising temperature variations, however, extreme fluctuations in rainfall could create significant harvest losses^13^.

Severity of climate impact on yield differ by crop, geographical location, and magnitude, as well as the direction of shift in the climatic variables^19,20^. Worldwide climate change impacts are uneven, particularly in nations with vast land areas^21,22^. Scientific community agrees to some extent that the present trends would be detrimental to the tropical and subtropical areas of Africa, middle east, south, and southeast Asia^23–26^, and advantageous to Russia, Ireland^27^, Canada^28,29^, and Finland^30,31^ in the context of cereal yield. Similarly, scientists have mixed opinions on climate change impact on US cereal production where Adams et al.^32^, Knox et al.^33^, Wolfe et al.^34^, and Petersen^35^, deduced it to be beneficial, and Schlenker et al.^23^, NDRC^24^, You et al.^25^, Raza et al.^36^ and Su et al.^20^ to be detrimental.

Farming in the SE-US may be highly susceptible to changing climate. Prevailing summer daily maximum temperature (T_max_) in this region frequently surpasses 32 °C, evaporation outpaces cropping period rainfall, and soils have poor water retention. The viability of agribusiness in the SE-US is dependent on lower capital inputs, eliminating certain choices in reducing the effects of changing climate^37^. Even though numerous past studies using different crop circulation models have already measured the potential climatic scenarios affecting crop yields at the global scale, regional level inferences, particularly in the SE-US, and on cereal crops remain under-researched^38^. As a result, the difficulties, and benefits to producers in the SE-US region remain unknown. Therefore, the objective of this study was to investigate and quantify the impact of climate changes (rainfall, T_max_, and T_min_,) in the previous 41 years on corn, rice, and wheat yields in SE-US.

## Material and methods

### Region and timespan of study

The SE-US is among the most diverse agro-ecological region, with an economy that largely relies on agriculture^39,40^. A total of 15.7% of the land area of the SE-US is dedicated to crop production and it constitutes about 13% of total US agricultural land^41^. Owing to its latitudinal, topographical, and geographical position relative to the Gulf of Mexico and the Atlantic Ocean, the SE-US is overly sensitive to extreme occurrences i.e., rising sea levels, hurricanes, heat waves, and dry spells, which further aggravates the nocturnal and diurnal temperature peaks^42^. These extreme events or natural disasters occur more frequently in the SE-US than in other parts of the nation altogether^43,44^. Furthermore, the SE-US groundwater resources are stressed owing to seasonal water scarcity and are expected to worsen by 2050, impacting agricultural production and forestry^45^.

The study utilized recent 41 years (1980–2020) of data. Generally, a minimum 30-year is required to sufficiently capture climate variations^46^. As of 1970 in the SE-US, the incidences of average days with temperatures exceeding 95°F and nights above 75°F have increased, while the prevalence of exceptionally cold days has decreased^47^. Moreover, the study encompassed 1983–2012 period during which the northern hemisphere witnessed the warmest 30 years stretch in the last 800 years^48^.

### Data

This study utilized a panel dataset commonly used in literature^49–54^ to predict the effect of climate change on cereal crop yield. In the panel dataset, the cross-sectional data is spread over a continuous time series^40,55^. The T_max_, T_min_, daily average temperature (T_avg_), rainfall, and crop yields represent cross-sectional data, and the years (1980–2020) represent the time-series data to complete a panel data set of 451 (row-wise) observations (41 years of data from 11 states), and 15 (T_max_, T_min_, T_avg_, rainfall, and yields × 3 crops) column-wise observations in a fixed-effect model.

**Figure 1:**
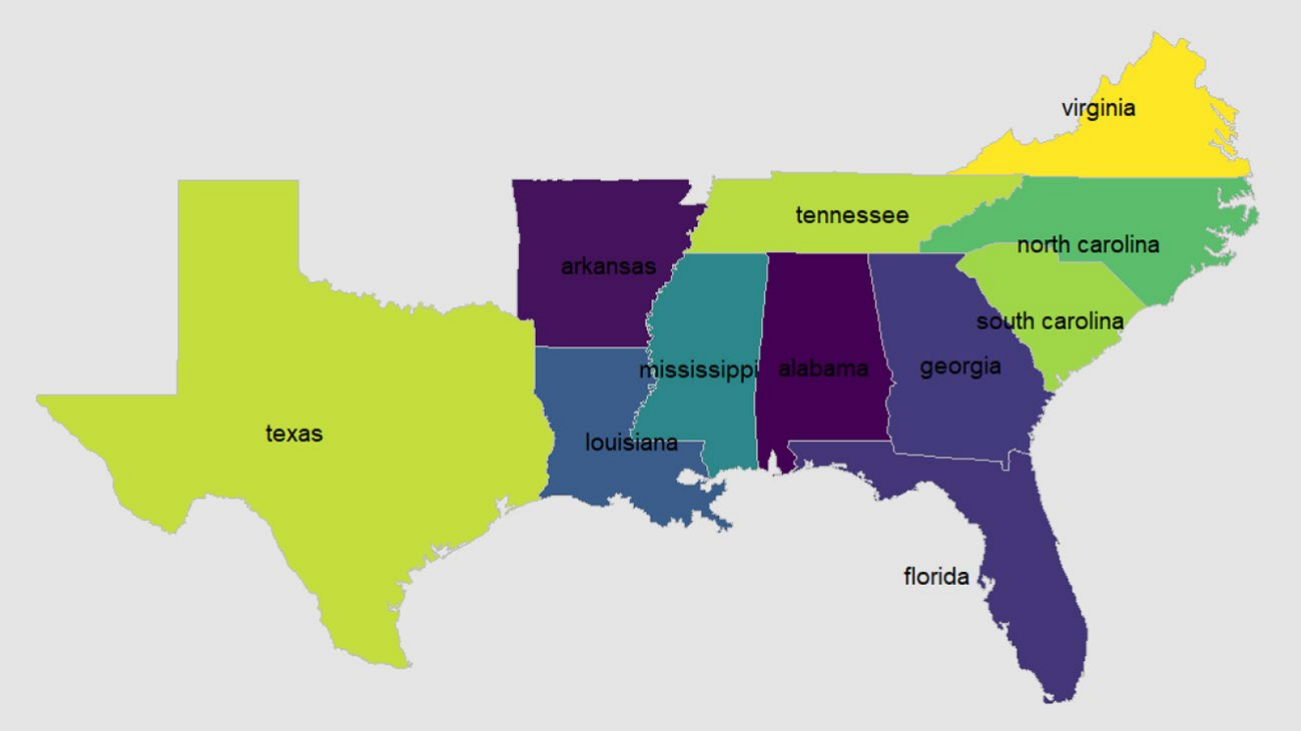
The map showing 11 states of SE-US considered in this study. Figure created using RStudio 2022.07.1, https://www.rstudio.com/.

The explanatory variables were rainfall, T_max_, and T_min_, and the response variables were corn, rice, and wheat yield from the past 41 years (1980–2020) of 11 states of the SE-US region (Table 1; Fig. 1). The yield statistics for each crop were derived from the National Agricultural Statistics Service's repository^56^. The county based daily weather data for all states from 1980 to 2020 for each month were collected by accessing US Climate Divisional Database^57^. This daily weather (T_max_, T_min_, and rainfall) data from all counties where respective crops are grown were averaged. The data source^57^ calculates county values by area-weighted mean of grid point observations transcoded from monitoring stations. A nominal 5-km grid-resolution was adopted to confirm spatial sufficiency in sampling.

**Table 1:** Description of the explanatory and response variables used in the fixed effect panel model.

Crops	Period	Panel districts	No. of years	Variables considered
Wheat	September to May	Alabama Arkansas Georgia Louisiana Mississippi North Carolina South Carolina Tennessee Texas Virginia	41 years (1980–2020)	T_max_ (°C) T_min_ (°C) T_avg_ (°C) Rainfall (mm) Wheat yield (Mg ha^−1^)
Rice	April to September	Arkansas Louisiana Mississippi Texas Virginia	41 years (1980–2020)	T_max_ (°C) T_min_ (°C) T_avg_ (°C) Rainfall (mm) Rice yield (Mg ha^−1^)
Corn	March to September	Alabama Arkansas Florida Georgia Louisiana Mississippi North Carolina South Carolina Tennessee Texas Virginia	41 years (1980–2020)	T_max_ (°C) T_min_ (°C) T_avg_ (°C) Rainfall (mm) Corn yield (Mg ha^−1^)

The data of explanatory variables were collected for all 11 states in the SE-US, however, the response variables (crop yields) data was collected for the states listed in the respective panel districts column in Table 1. All states that had their continuous yield statistics available on the USDA-NASS website from 1980 to 2020 for the required crops (corn, rice, and wheat), were grouped together to form crop-specific panel districts mentioned in Table 1. The fixed-effect model is not applicable to non-continuous datasets^58^, as such the entire data was ensured to be continuously consistent from 1980 to 2020. The corn growing season (CGS) was from March to September, wheat growing season (WGS) from September to May, and rice-growing season (RGS) from April to September, respectively, as per the agricultural handbook of USDA on sowing and harvesting dates for field crop^56^. The daily temperatures for each crop were converted into the average growing period temperature, and the daily rainfall was summed to cumulative total rainfall for each crop growing period similar to the calculations suggested by others^51,59^.

The results of the collinearity test among explanatory variables are shown in Table 2. Variance inflating factor (VIF) less than 10 indicates no collinearity among covariances in each crop^60^.

**Table 2:** Multicollinearity statistics.

Particulars	VIF	SQRT VIF	Tolerance
WT (min)	12.49	3.53	0.08
WT (max)	13.27	3.64	0.08
WR	1.91	1.38	0.52
Mean VIF	9.22		
RT (min)	7.43	2.73	0.13
RT (max)	5.75	2.40	0.17
RR	3.87	1.97	0.26
Mean VIF	5.68		
CT (min)	13.93	3.73	0.07
CT (max)	12.10	3.48	0.08
CR	2.96	1.72	0.34
Mean VIF	9.66		

WT, RT, and CT represent the Wheat temperature, Rice temperature, and corn temperature, respectively. WR, RR, and CR represent the Wheat rainfall, Rice rainfall, and Corn rainfall, respectively. VIF represents Variance inflating factor, and SQRT VIF is square root of VIF.

### Panel data approach and analysis

The panel data analysis is an accepted approach to assess the impact of temperatures and rainfall on crop yields and is widely used^61–68^. The panel-data model is regarded as superior to other econometric models, and is robust in the context of heterogeneity verification, increasing the degrees of freedom, and decreasing correlations between unobserved factors affecting the response variable, yield^69,70^.

Under the panel-data approach, either a random effect model or a fixed-effect model is generally considered. Our study utilized a fixed-effect model to account for the relationship between regressors (crop yields) and the time-independent distinctions of unobserved variables^61,70^. These time-independent parameters include soil features, topographic factors, and farmers' self-governing adjustments, for example, altering planting time or cultivars, and varying input amounts due to yearly variations in meteorological parameters^51,71^. Contrarily, the random-effects model indicates no relation of time-independent attributes with explanatory factors^72^. Fixed-effect model has also been supported by subsequent relevant studies^73,74^. Panel analysis of data quantifies the impact of climate on agricultural output by calculating a production function using regression^75^. Empirical estimates of such functions, on the other hand, are centered on a panel set of data, which comprises an observational dataset in a single component cross-sectional unit (corn, rice, and wheat yields)^76^. The spatially fixed model effects in the panel dataset absorb the region-specific time-dependent determinants of agricultural yields, which might be associated with meteorological variables^51^. Rainfall and temperatures (T_max_ and T_min_) are recognized to be the key factors for crop yield. Hence, the fixed effects panel model used for climate effects in the present study is as follows:

1 $$\mathit{ln}{y}_{it}={S}_{i}+{T}_{t}+{\beta X}_{it}+{\varepsilon }_{it}$$

The states are denoted by *i* and the time is denoted by *t* in the above equation. The crop yield in the model is the response variable which is denoted by *y*, and the fixed effects of the state are denoted by *S*. The study has a hypothesis that the state fixed effects (*S*) incorporate all unconsidered state-specific characteristics that change over time, affect yields, and reduce noise caused by extraneous variables in the study model. The yield estimation model has *T* to symbolize time fixed effects, which might be caused by infrastructural advancement factors, changes in technologies, human assets improvements, etc. The climatic factors are denoted by *X*, whereas *β* is related to explanatory variable parameters, and ϵ is the random term.

Then, the panel data of T_max_, T_min_, rainfall, and crop yields were analyzed using Stata® version-16 statistical software^77^. We calculated the magnitude and rate (per year) of change (Table 4) that occurred in climatic variables from 1980 to 2020, during each crop growing season. The annual rate of change is important, as a greater value of climatic variables allows a shorter time for ecosystems for readjustment^78^.

Each crop yield was separately regressed on positive variation in climatic factors during its growing season, and the respective regression coefficients with *p* values were calculated (Table 5). These coefficients revealed the exact change (increase/decrease) in crop yields due to changes in climatic variables. A series of studies conducted by Schlenker and Roberts^79^, Guiteras^80^, Jacoby et al.^81^ and Birthal et al.^62^ revealed that the effect of temperature and rainfall on crop yield is generally non-linear. Hence, the squared factor of each climatic variable was introduced along with the climatic variables in the equation to account for this non-linearity issue^82^. These squared terms caused the inordinate variability in yield (*y*_*it*_). The Eq. (1) was transformed to log-linear (logarithmic function) to control the large variability in *yit*. The coefficients of the log-linear function can be easily interpreted as proportionate changes using marginal effects. Therefore, the marginal effects of temperature and rainfall were determined by calculating the net response of crop yields to climatic factors equating mean average values for each variable in the equation. Then, the net change in crop yield by a 10 mm shift in rainfall and a 1 °C shift in temperatures (T_max_ and T_min_) was mathematically derived.

To determine the pattern in T_max_, T_min_, and rainfall throughout the crop growing seasons, the log (natural) values of these variables were regressed over time by applying district (state) fixed effects to control the time impendent parameters^62^.

### Diagnostic testing

Before applying regression to the fixed-effect model for estimation, a sequence of diagnostic procedures was performed to test assumptions of autocorrelation among individual time-independent attributes, by the model's error components. Since every entity is unique, its error, as well as constant term, must be uncorrelated. If error terms correlate, the fixed-effect model is inapplicable, and inferences drawn would be false. There is a chance of non-stationarity with the response (*y*_*it*_) and explanatory variables (temperatures and rainfall) that could lead to an autocorrelation problem, which is a more serious issue with the explanatory variables.

We used the panel unit root tests, such as Levin, Lin, and Chu^83^; Im, Pesaran, and Shin^84,85^ and Fisher-type tests^86^, for testing the stationarity and rejected the null hypothesis for all the series (Table 3). Conclusively, all meteorological variables in the datasets were stationary, and the problem of autocorrelation was non-significant in the data.

**Table 3:** Various tests to check the stationarity in the data.

Variables	Levin-Lin-Chu			Im-Pesaran-Shin		Fisher-type	
	Unit root test			Unit root test		Unit root test	
	Unadjusted t	Adjusted T	*p* value	z-t-tilde-bar	*p* value	Chi-sq (pm)	*p* value
	H_0_: Panel contains unit root			H_0_: All panel contain unit roots		H_0_: All panel contain unit roots	
	H_1_: Panel are stationary			H_1_: Some panels are stationary		H_1_: At least one panel is stationary	
WT (min)	− 12.331	− 8.590	0.00	− 7.806	0.00	139.966	0.00
RT (min)	− 6.8925	− 4.853	0.00	− 4.456	0.00	46.886	0.00
CT (min)	− 9.8770	− 6.606	0.00	− 7.136	0.00	122.845	0.00
WT (max)	− 15.165	− 11.810	0.00	− 9.175	0.00	188.179	0.00
RT (max)	− 9.153	− 6.783	0.00	− 5.856	0.00	76.669	0.00
CT (max)	15.791	− 11.738	0.00	− 10.764	0.00	272.529	0.00
WR	− 15.482	− 10.00	0.00	− 11.396	0.00	322.525	0.00
RR	− 7.962	− 4.828	0.00	− 6.606	0.00	108.058	0.00
CR	− 14.602	− 9.118	0.00	− 12.089	0.00	369.165	0.00
Ln (yield wheat)	− 6.019	− 3.133	0.00	− 6.819	0.00	127.437	0.00
Ln (yield rice)	− 2.982	− 1.945	0.00	− 1.181	0.10	10.465	0.23
Ln (yield corn)	− 6.726	− 3.499	0.00	− 7.486	0.00	152.610	0.00

WT, RT, and CT represent the Wheat temperature, Rice temperature, and Corn temperature, respectively. WR, RR, and CR represent the Wheat rainfall, Rice rainfall, and Corn rainfall, respectively.

## Results and discussions

The climatic variations, their effect along with their marginal effect on crop yield are discussed under three different sections i.e., corn, rice, and wheat as follow.

### Corn

#### Changes in climatic variables during CGS

**Figure 2:**
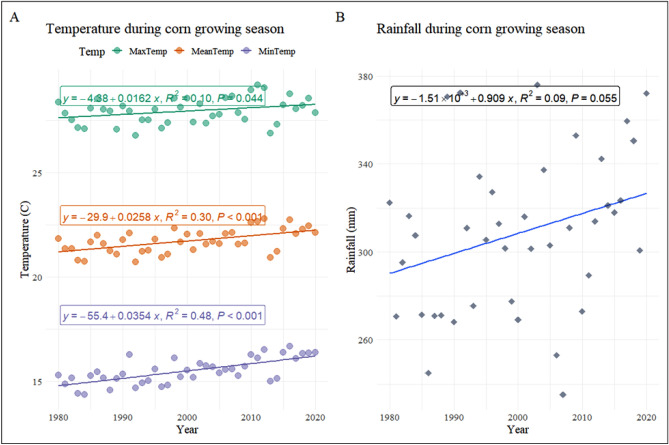
(**A**) T_max_, T_min_, and T_avg_, showed significant slopes throughout CGS in the SE-US between 1980 and 2020. (**B**) The rainfall showed a non-significant trend in the SE-US over CGS between 1980 and 2020.

T_max_, T_min_, T_avg_, and rainfall (Table 4) averaged 28 °C, 15.50 °C, 21.70 °C, and 308 mm over the 41-year period, respectively. Between 1980 and 2020 during CGS, T_max_, T_min_, T_avg_, and rainfall had an increasing trend, however, slope was significant for temperatures only (Fig. 2), and all shifted by 0.64 °C, 1.40 °C, 1.02 °C, and 36.3 mm, respectively (Table 4).

**Table 4:** Overall mean, change and the annual rate of change in temperature and rainfall during different crop growing seasons in the SE-US, 1980–2020.

Particulars	T_max_ (°C)	T_min_ (°C)	T_avg_ (°C)	Rainfall (mm)
**Wheat**				
Mean	20.0 (0.002)	7.3 (0.002)	13.7 (0.002)	369 (0.027)
Change	1.12	1.48	1.30	19.2
Annual rate of change	0.028***	0.037***	0.033***	0.48***
**Rice**				
Mean	28.9 (0.005)	16.4 (0.003)	22.7 (0.004)	292 (0.035)
Change	0.64	1.28	0.96	37.2
Annual rate of change	0.0158	0.033	0.024	0.93
**Corn**				
Mean	28.0 (0.003)	15.5 (0.002)	21.7 (0.003)	308 (0.021)
Change	0.64	1.40	1.02	36.3
Annual rate of change	0.017***	0.035***	0.026***	0.91***

***Denote significance at 1% level, Figures in parentheses are standard errors.

During CGS, T_min_ and T_max_ contributed 68.63% and 31.37%, respectively, to overall warming (Table 4), indicating that the nocturnal temperature explains the majority of the CGS heating trend, consistent with findings of Peng et al.^87^ and Screen^88^. This overall warming could be advantageous or deleterious to crops depending on the growth stage of the crop when it occurs^89^. For example, high temperature during initial reproductive or delayed vegetative corn stages reduce the ripening span, stress the plant, and decrease the overall yield^90^. T_min_ (0.035 °C per year) changed at a faster annual rate than T_max_ (0.017 °C per year) and T_avg_ (0.026 °C per year), indicating that the nocturnal warming rate (annual) was 105.90% quicker than the diurnal warming (Table 4). A similar trend in warming was documented in other parts of the US corn belt^91^, and is continuously progressing with time^92–97^.

#### Impact of climate change on corn yield

The estimated T_max_ and T_min_ regression coefficients were significant, indicating temperature to be the major variable affecting corn production in the SE-US (Table 5). The T_max_ exhibited a significantly negative regression coefficient meaning a deleterious effect on corn yield (Table 5). Similar results for corn were noted by Stooksbury and Michaels^98^, Mourtzinis et al.^90^, and Eck et al.^99^ in the SE-US. The increased T_max_ stimulates water stresses (up to 60%), decreases photosynthetic activity, and negatively affects the antioxidant enzyme in the corn plant^100–102^. Moreover, the average T_max_ of CGS was 28 °C (Table 4) which is greater than the optimum temperature (26.40 °C) for corn^103^ and is approaching 29 °C which could be detrimental to corn^104^. The regression coefficient for T_min_ was significantly positive (Table 5), implying that T_min_ increased corn yield. Similar effects were documented by Stooksbury and Michaels^98^ in the SE-US, and Chen et al.^105^. Increased T_min_ increases corn kernel weight by remobilizing the stored dry matter from other parts of the plant^106^. The overall impact of incremental changes in T_max_ and T_min_ was still beneficial to the corn yield, which may be statistically inferred as every 1 °C increment in net temperature enhances corn yield. The positive effects of T_min_ compensated for the negative effects of T_max_ on corn, resulting in an overall yield increase. These findings agree with Ruane et al.^107^, Kukal et al.^108^, Petersen^35^, and Ding and Shi^109^, but contradict Lin et al.^110^, and Chen et al.^111^. Disagreement in the literature could be attributable to a number of factors associated with different studies such as different study periods taken, spatial diversity (different growing seasons), the magnitude of change in climatic variables, diversity in crop models, and statistical approaches^112–116^. Another explanation as per Lobell et al.^61^, is that if the T_avg_ of CGS is below the optimum (23 °C), the overall influence of temperature warming will increase yield, and above 23 °C, the yield will drop. In our study T_avg_ was 21.70 °C (Table 4).

**Table 5:** Regression estimates of the impact of temperature and rainfall on the yield of major cereal crops in the SE-US, 1980–2020.

Particulars	Wheat			Rice			Corn		
	C	SE	*p* value	C	SE	*p* value	C	SE	*p* value
T_min_ (°C)	− 0.01	0.08	0.89	0.18***	0.29	0.01	0.39***	0.18	0.01
T_min_ (Square)	0.00	0.00	0.51	0.00	0.01	0.88	0.00	0.01	0.67
T_max_ (°C)	0.14	0.19	0.45	− 1.09***	0.38	0.01	− 1.12***	0.30	0.01
T_min_ (Square)	0.00	0.00	0.58	0.02***	0.01	0.01	0.01***	0.01	0.01
RF (mm)	− 0.03***	0.01	0.01	0.01	0.01	0.41	− 0.01	0.02	0.88
RF (Square)	− 0.0004***	0.00	0.00	0.00	0.00	0.22	− 0.0003	0.00	0.24
Constant	1.33	1.77	0.45	22.10	5.11	0.00	18.49	3.65	0.00
District		Yes			Yes			Yes	
Time		Yes			Yes			Yes	
No of observations		410			164			451	

***Denote significance at the 1% level; C, SE, and RF represent regression coefficient, standard error, and rainfall, respectively.

The rainfall regression coefficient (Table 5) and marginal effect (Table 6) were found to be non-significant for corn and the same was noted by Lobell et al.^117^ and Guntukula^118^. Most of the corn cultivation in the SE-US is based on irrigated systems^119^, which, according to Chen et al.^105^, may be the reason for the weak relationship of rainfall with corn yield in most studies.

**Table 6:** Marginal effects of climate change on major cereal crop yields in the SE-US, 1980–2020.

Particulars	Wheat			Rice			Corn		
	C	z-value	*p* value	C	z-value	*p* value	C	z-value	*p* value
T_min_ (°C)	− 0.04	− 1.27	0.20	0.224***	6.87	0.01	0.47***	13.46	0.01
T_max_ (°C)	0.04	1.44	0.15	− 0.083***	− 2.48	0.01	− 0.34***	− 9.15	0.01
Rainfall (mm)	− 0.0009***	− 0.39	0.01	0.002	− 0.61	0.542	− 0.01	− 3.55	0.21

***Denotes significance at the 1% level; C represents the regression coefficient of marginal effects.

#### Marginal impact of climate change on corn yield

The marginal coefficient of regression (Table 6) for T_max_ was − 0.34 (significant), implying a 34% yield reduction for every 1 °C increase in T_max_. Others have noted a reduction of up to 10%^120^, 15%^90^, and 30%^121^, or even up to 80% in worst scenarios^122^. The T_min_ (Table 6) marginal regression coefficient was found to be 0.47 (significant), indicating that every 1 °C increase in T_min_ increased corn yield by 47%. T_min_ never reached the threshold that could have shifted T_avg_ (Table 4) out of the corn optimal range of 20–30 °C, reducing corn yield^106,123^. Therefore, corn benefited comparatively more from the increase in T_min_. Moreover, T_min_ is of greater importance compared to T_max_ in governing yield-determining developmental and grain filling processes^124,125^, and T_min_ impact has a comparatively higher magnitude (47% > 34%) in our study results (Table 6). Consequently, the magnitude of the positive effect of T_min_ surpassed the negative effect of T_max_ implying that each 1 °C increase in net temperature resulted in a 13% increase in corn yield (Table 6) which is in line with Zhang et al.^126^ who documented the overall positive effect of an increase in net temperature on corn yield.

### Rice

#### Changes in climatic variables during RGS

**Figure 3:**
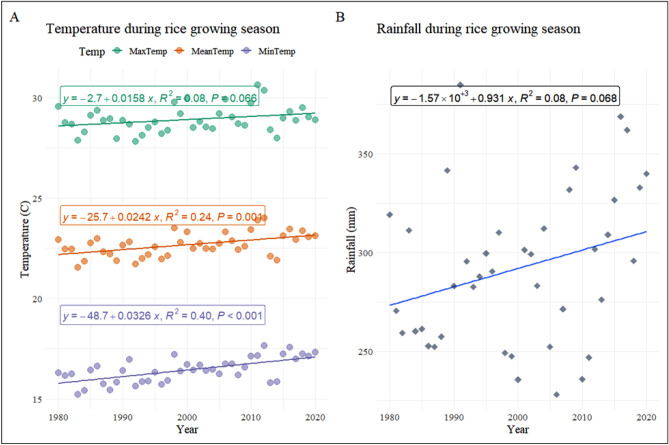
(**A**) T_max_ showed a non-significant slope whereas T_min_, and T_avg_, showed significant slopes throughout RGS in the SE-US between 1980 and 2020. (**B**) The rainfall showed a non-significant trend in the SE-US over RGS between 1980 and 2020.

Over the 41-year period, the average values (Table 4) for T_max_, T_min_, T_avg_, and rainfall were 28.90 °C, 16.40 °C, 22.70 °C, and 292 mm during RGS. T_max_, T_min_, T_avg_, and rainfall have all shifted (Table 4) by 0.64 °C, 1.28 °C, 0.96 °C, and 37.2 mm following an increasing trend (Fig. 3) between 1980 and 2020.

The increase in T_max_ (0.64 °C) and T_min_ (1.40 °C) noted during RGS in the SE-US (Table 4) were higher than the global T_max_ (0.40 °C) and T_min_ (0.80 °C) increases^9,127^. In rice, the reproductive phase is undoubtedly more vulnerable than the vegetative phase to these increased temperatures^103,128^. T_min_ and T_max_ contributed 66.70% and 33.30%, respectively, to total warming (Table 4), and a similar asymmetric warming trend was previously confirmed by Donat and Alexander^129^ and Peng et al.^95^. Overall, the T_min_ describes most of the RGS heating trends in SE-US. Rainfall has changed by 0.93 mm per year (Table 4). The yearly rate of change of T_min_ (0.033 °C per year) was greater than the rates of change of T_max_ (0.016 °C per year) and T_avg_ (0.024 °C per year), implying that the nocturnal warming (T_min_) was 106.30% quicker than diurnal warming (T_max_). These annual rates of increase are unproblematic until they can keep the resulting temperature within the optimal range and below the extreme cardinal value (35 °C)^87,130^. These optimum temperature ranges for rice are 30–32 °C as per TNAU^131^ and 22–31 °C as per Yoshida^132^.

#### Impact of climate change on rice yield

The computed T_max_ and T_min_ regression coefficients for rice indicated that diurnal and nocturnal temperatures are the most important variables in rice production (Table 5). The calculated regression coefficient for T_max_ was negative (significant) (Table 5), implying a decrease in rice yield by every 1 °C rise in T_max_. Zhang et al.^114^, Dubey et al.^133^, and Guntukula^118^ reported similar results where increased T_max_ lowered rice yield by increasing spikelet sterility. The rise in T_max_ causes increased plant respiration, evapotranspiration, plant water, and nutrient losses, and decreased crop durations, leading to lower water and nutrient use efficiency in rice^9,13^. Oh-e et al.^134^ concluded that any additional increase in mean T_max_ above 28 °C could diminish rice yields, and this study noted T_max_ (Table 4) to be 28.90 °C (> 28 °C), negatively influencing rice yield. The T_min_ coefficient of regression was significantly positive (Table 5), indicating a significant increase in rice yield for every 1 °C increase in T_min_, supporting the findings of Zhang et al.^114^, Guntukula^118^, and Tan et al.^65^, but contradicting Zhang et al.^135^ and Ghadirnezhad and Fallah^136^. However, Cooper et al.^137^ found no change in rice yield with rising T_min_. According to Agrawal et al.^138^, the increased T_min_ had a positive effect over the early, delayed vegetative, or reproductive phases, and a negative effect throughout the ripening phase. Moreover, according to Mohammed and Tarpley^139^, and Nagarajan et al.^140^, increased T_min_ between 21 and 32 °C has a negative impact on the plant respiratory system, reducing rice yield, but SE-US’s T_min_ average (16.40 °C) (Table 4) was outside this range, and not even hit the threshold level to start impacting rice yields negatively. Therefore, this study revealed that increasing T_max_ and T_min_ has a net beneficial effect on rice yield, as indicated by the fact that every 1 °C overall increase in temperature improves rice grain yield. The positive effect of T_min_ outpaced the negative effect of T_max_ on rice yield, increasing the rice yield. Similar findings showing a net beneficial effect of changing temperatures on rice yield were reported by Kim and Pang^141^, Petersen^35^, and Ding and Shi^109^.

Rainfall increments numerically improved rice yield but were not statistically significant (Table 5). Despite rice's water sensitivity, the impact of rainfall on rice yield was statistically insignificant because most of the rice is grown on assured irrigation systems in the SE-US^119^.

#### Marginal impact of climate change on rice yield

The marginal regression coefficient (Table 6) for T_max_ in rice was − 0.083 (significant), indicating a 1 °C surge in T_max_ significantly decreased rice yield by 8.30%. Contrarily, every 1 °C rise in T_min_ significantly increased rice yield by 22.40% since the marginal coefficient for T_min_ was computed as 0.224 (significant). Consequently, the net marginal effect of both T_max_ and T_min_ increased the rice yield by 14.10%. These findings are in line with the results of Kim and Pang^141^, who documented a 10–20% increase in rice yield, whereas Saseendran et al.^142^ calculated only a 6% net increase. Although statistically insignificant every 10 mm rise in rainfall was found to increase the rice yield by 0.20%.

### Wheat

#### Changes in climatic variables during WGS

**Figure 4:**
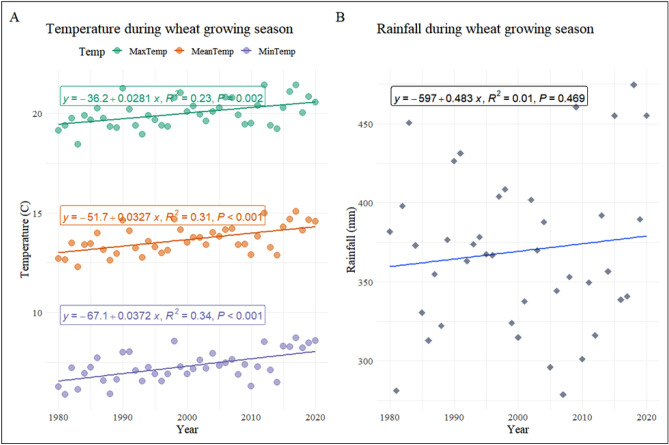
(**A**) T_max_, T_min_, and T_avg_, showed significant slopes throughout WGS in the SE-US between 1980 and 2020. (**B**) The rainfall also showed a non-significant trend in the SE-US over WGS between 1980 and 2020.

The average values for T_max_, T_min_, T_avg_, and rainfall were noted as 20 °C, 7.30 °C, 13.70 °C, and 369 mm over the 41-year timespan (Table 4). From 1980 to 2020, the T_max_, T_min_, T_avg_, and rainfall followed a significant increasing trend (Fig. 4) and shifted by 1.12 °C, 1.48 °C, 1.30 °C, and 19.2 mm (Table 4).

These shifts in warming are comparable with those experienced in other parts of the US^143^ and worldwide^144^. T_min_ and T_max_ contributed 56.92% and 43.08%, respectively, to overall warming throughout the 41-year period of WGS. This warming could benefit wheat yields in certain environments, but it may diminish yield in areas where optimum temperatures already prevail^145^. The annual rate of change of T_min_ (0.037 °C per year) was greater than the rate of change of T_max_, (0.028 °C per year) and T_avg_ (0.033 °C per year), however, the rainfall changed by 0.48 mm per year. The T_min_ is increasing at a 32.14% faster rate than T_max_, implying an unsymmetrical warming trend of 32.14% quicker nocturnal warming than the diurnal warming during WGS. Dhakhwa and Campbell^101^ noted that asymmetric warming may have a less devastating impact on yield than uniform warming.

#### Impact of climate change on wheat yield

The T_max_ showed a non-significant but numeric yield gain during WGS. These results are in parallel with the findings of Zhang et al.^146^, and Fang et al.^147^ who inferred positive effects of T_max_ on wheat yield. Past studies also showed a weak relationship (insignificant) of T_max_ with the wheat yield^148^. Normally, T_max_ above 32 °C during grain filling has a negative impact on wheat yield^147^ but this has not been the case with our study, because in the SE-US, these elevated T_max_ values are likely to occur in July and August and wheat harvesting season ends in May. Although the coefficient of regression (Table 5) for T_min_ during WGS was also noted to be statistically insignificant, T_min_ numerically decreased wheat yield. Similarly, past studies^147^ also deduced that T_min_ did not affect wheat yield, but numeric yield reduction was noted by Prasad et al.^149^. Moreover, some of the estimated T_max_ and T_min_ weaknesses (statistical insignificance) are due to fluctuation during the months of October, November, and December in WGS, not present for maize and rice. The results reported no change in wheat yield by the net effect of T_max_ and T_min_ in the SE-US during WGS over the studied period. Rainfall significantly reduced wheat yield (Table 5), which means that for every 1 mm increase in rainfall, wheat grain yield decreased in SEUS.

#### Marginal impact of climate change on wheat yield

Table 6 indicates that the marginal regression coefficient for T_min_ was − 0.04 implying a 4% reduction in wheat yield with every 1 °C rise in T_min_. T_max_, on the other hand, was 0.04 indicating that every 1 °C rise enhanced yield by 4%. Despite producing statistically insignificant results, the equation's robustness, and coefficient’s strength (Table 6) were found to be better in the case of T_max_ compared to T_min_, indicating that T_max_ is comparatively more associated with wheat yield than T_min_. This is in line with the study of Jha and Tripathy^150^ who also concluded T_max_ to be more impactful than T_min_ on wheat yield. The results had shown that there was no change in final wheat yield due to the net effect of T_max_ and T_min_.

Rainfall significantly affected wheat yield negatively, but the effect was meager only a 0.09% decrease in wheat yield with every 10 mm rainfall increment as per the marginal regression coefficient (− 0.0009). These results are in line with the findings of Bhardwaj et al.^151^, Ureta et al.^152^, and Guntukula^118^ who also realized a decrease in wheat yield due to an increase in rainfall.

Furthermore, it is suggested to explore the similar impacts targeting different growth stages of cereals for a more detailed understanding of how this impact varies with the different growth stages for each crop. A more detailed county-wise study for each state could also generate a better understanding of the SE-US agro-climatic scenario.

## Conclusion

The results of fixed-effect model revealed a significant temporal variability in rainfall and temperatures across the SE-US, and an asymmetrical pattern of nocturnal and diurnal warmings throughout the CGS, RGS, and WGS. T_min_ contribution was higher during CGS (68.63% > 31.37%), RGS (66.70% > 33.30%), and WGS (56.92% > 43.08%) than T_max_ in overall warming. Furthermore, the rate of increasing T_min_ was noted to be 105.90%, 106.30%, and 32.14% higher than the T_max_ during CGS, RGS, and WGS, respectively. During CGS, RGS, and WGS, rainfall had shifted by 36.3 mm, 37.2 mm, and 19.2 mm, with annual rates of change of 0.91 mm/year, 0.93 mm/year, and 0.48 mm/year, respectively. Rainfall had a negative (non-significant), positive (non-significant), and negative (significant) effect on corn, rice, and wheat yields, respectively. Overall, climate change in the SE-US had no net effect on wheat yield but significantly increased corn yield by 13%, and rice yield by 14.10%.

## Data Availability

The data used is collected from National Agricultural Statistics Service's repository (USDA-NASS) and US Climate Divisional Database, (NOAA).
